# A cross-sectional survey of prevalence and correlates of suicidal ideation and suicide attempts among prisoners in New South Wales, Australia

**DOI:** 10.1186/1471-2458-12-14

**Published:** 2012-01-06

**Authors:** Sarah Larney, Libby Topp, Devon Indig, Colmán O'Driscoll, David Greenberg

**Affiliations:** 1 Centre for Health Research in Criminal Justice, Justice Health and Forensic Mental Health Network, Sydney, Australia; 2 National Drug and Alcohol Research Centre, University of New South Wales, Sydney, Australia; 3 The Kirby Institute (formerly the National Centre in HIV Epidemiology and Clinical Research), University of New South Wales, Sydney, Australia; 4 School of Public Health and Community Medicine, University of New South Wales, Sydney, Australia; 5 Statewide Forensic Mental Health, Justice Health and Forensic Mental Health Network, Sydney, Australia; 6 School of Psychiatry, University of New South Wales, Sydney, Australia

## Abstract

**Background:**

We aimed to estimate the prevalence of suicidal ideation and suicide attempt among prisoners in New South Wales, Australia; and, among prisoners reporting suicidal ideation, to identify factors associated with suicide attempt.

**Methods:**

A cross-sectional design was used. Participants were a random, stratified sample of 996 inmates who completed a telephone survey. The estimated population prevalence of suicidal ideation and suicide attempt were calculated and differences by sex and Aboriginality were tested using *χ*2 tests. Correlates of suicidal ideation and suicide attempt were tested using logistic regression.

**Results:**

One-third of inmates reported lifetime suicidal ideation and one-fifth had attempted suicide. Women and Aboriginal participants were significantly more likely than men and non-Aboriginal participants, respectively, to report attempting suicide. Correlates of suicidal ideation included violent offending, traumatic brain injury, depression, self-harm, and psychiatric hospitalisation. Univariate correlates of suicide attempt among ideators were childhood out-of-home care, parental incarceration and psychiatric hospitalization; however, none of these remained significant in a multivariate model.

**Conclusions:**

Suicidal ideation and attempts are highly prevalent among prisoners compared to the general community. Assessment of suicide risk is a critical task for mental health clinicians in prisons. Attention should be given to ensuring assessments are gender- and culturally sensitive. Indicators of mental illness may not be accurate predictors of suicide attempt. Indicators of childhood trauma appear to be particularly relevant to risk of suicide attempt among prisoners and should be given attention as part of risk assessments.

## Background

Suicide in prison is a major concern, occurring at 3-8 times the rate seen in the general community [1]. Risk factors for suicide, such as mental illness, substance use disorders and impulsivity are highly prevalent among prisoners [2-4], and inmates import these risk factors into the correctional setting [5]. The powerlessness and social exclusion experienced in prison may also contribute to suicidal behaviour [6,7].

Prison suicides are associated with many of the same risk factors as seen in suicides more generally, such as prior suicidal ideation and suicide attempts; mental illness; substance use disorders; and self-harming behaviours [3,7-12]. Suicide risk factors specific to correctional settings have also been identified, such as unsentenced status (i.e. on remand) [3,13]; housing in single-occupancy cells [3]; and conviction for a violent offence [14]. The initial stages of incarceration are a period of particularly high suicide risk; in the United Kingdom and Australia, one in ten completed suicides in prison occur within the first twenty-four hours of imprisonment, and one-third within the first week [7,12]. The acute intoxication effects of alcohol and other drugs, such as reduced inhibitions and increased impulsivity, may contribute to suicide risk during this time [7,15].

Of particular concern in the Australian context is suicide among Aboriginal inmates. Aboriginal and Torres Strait Islander persons make up 2.5% of the Australian population, but 26% of prisoners, and are incarcerated at 14 times the rate of non-Indigenous persons [16,17]. Indigenous Australians have a significantly higher suicide rate than non-Indigenous persons [18]; however, a previous study of New South Wales (NSW) prisoners found no association between Aboriginality and a history of suicidal ideation or attempts [19]. To our knowledge, no other studies have examined correlates of suicidality among Australian prisoners, although a clinical audit found a high prevalence of established risk factors in completed suicides (e.g. substance use disorders; histories of self-harm; single cell accommodation) [12].

The high prevalence of suicide risk factors among prisoner populations means that these factors are of limited use in discriminating individuals at risk of suicide attempt [13,20]. As such, the clinical task of predicting and consequently preventing suicide attempts in prison remains problematic [20,21]. Given the close relationship between suicidal ideation and completed suicide among prisoners [3], understanding suicide risk in prison requires a greater understanding of the prevalence of suicide attempts in relation to suicidal ideation, and risk factors for suicide attempt in the presence of ideation. Therefore, this study aimed to:

1. Estimate the 12-month and lifetime population prevalence of suicidal ideation and attempt among prisoners in NSW;

2. Identify factors associated with lifetime suicidal ideation; and

3. Identify factors associated with lifetime suicide attempt among prisoners reporting lifetime suicidal ideation.

## Methods

Reporting of this study is in accordance with the Strengthening the Reporting of Observations Studies in Epidemiology (STROBE) statement [22].

Data were sourced from the 2009 Inmate Health Survey (IHS), a cross-sectional survey of the health and wellbeing of prisoners in NSW, Australia. NSW is Australia's most populous correctional jurisdiction, housing 37% of the country's prisoners [16]. Full methodological details for the IHS are provided elsewhere [23]. Briefly, between May 2008 and March 2009 a random sample of inmates stratified by age, gender and Aboriginal status was recruited from 30 adult correctional centres. Women and Aboriginal inmates were oversampled to allow credible estimates of low-prevalence conditions in these sub-populations. Inmates were excluded from participation if they did not speak sufficient English or had an intellectual disability or mental illness that prevented them from providing informed consent, or were aged under 18 years.

The survey instrument was administered via telephone, with interviewers entering inmate responses directly into a computer database. Participants were reimbursed AU$10 for their involvement. Approvals for the IHS were granted by the Justice Health Human Research Ethics Committee, the Corrective Services NSW Research Ethics Committee and the Aboriginal Health and Medical Research Council Ethics Committee.

### Measures

Suicidal ideation was assessed by asking participants "have you ever thought about suicide?". Participants who reported suicidal ideation were asked "When did you last think about suicide?". Suicide attempts were assessed by asking participants "have you ever thought about suicide?", with participants who responded yes asked "Thinking about your last suicide attempt, how long ago did this occur?". Participants did not need to endorse suicidal ideation to be asked if they had attempted suicide.

Variables explored for their relationship to suicidality were measured using standardised measures and stand-alone questions specific to this survey [23]. Aboriginal status was based on self-reported identification. Self-harm was defined as deliberately harming or injuring oneself, excluding suicide attempts. 'Regular illicit drug use' was defined as using illicit drugs on a daily or almost daily basis in the 12 months prior to incarceration. Traumatic brain injury (TBI) was defined as a head injury resulting in unconsciousness [24]. Participants scoring 17 or greater on the Beck Depression Inventory [25] were classified as having moderate/severe depression. The Alcohol Use Disorders Identification Test [26] was modified so as to refer to alcohol consumption in the 12 months prior to the current incarceration, with a score of 8 or more indicative of harmful alcohol use during this period.

### Statistical analysis

Statistical analyses were undertaken in SAS 9.2 [27]. Survey analysis procedures were used to adjust for the sample design. Sample weights were calculated as the inverse of the probability of being selected to participate in the survey and adjusted for non-response. The correctional centre in which the inmate was incarcerated was incorporated into analyses as a cluster variable to account for potential similarities between inmates housed in the same centre.

Weighted prevalence estimates of 12-month and lifetime suicidal ideation and suicide attempt were calculated for the total prison population and by gender and Aboriginality. Differences between groups were tested using *χ*^2 ^tests. Correlates of lifetime suicidal ideation were tested at the univariate level using logistic regression, with all variables significant at the *p *≤ .05 level entered into a multivariate logistic regression model. The same modelling strategy was used to examine correlates of lifetime suicide attempt among participants reporting lifetime suicidal ideation.

## Results

Of 1166 inmates randomly approached to participate, 996 agreed, yielding a response rate of 85.4%. Women comprised 20% (n = 199) of participants, and 31% (n = 312) of participants self-identified as Aboriginal.

### Prevalence of suicidal ideation and suicide attempts

The lifetime prevalence of suicidal ideation among NSW inmates was 33.7%, and of suicide attempt was 20.5% (Table 1). There was no gender difference in terms of prevalence of suicidal ideation; however, women were significantly more likely than men to report a lifetime suicide attempt (28.7% vs. 19.9%, *p *= .03). Similarly, there was no difference in suicidal ideation between Aboriginal and non-Aboriginal inmates; however, Aboriginal inmates were significantly more likely than non-Aboriginals to report a lifetime suicide attempt (26.9% vs. 18.7%, *p *= .01), and were more than twice as likely to have attempted suicide in the previous 12 months (4.4% vs. 2.1%, *p *= .03).

**Table 1:** 12-month and lifetime prevalence of suicidal ideation and suicide attempts among NSW inmates

	% (95% confidence interval)				
	
	Men	Women	Non-Aboriginal	Aboriginal	All persons
Suicidal ideation					

12-month	9.1 (6.6-11.7)	10.8 (5.6-16.1)	8.7 (5.8-11.5)	11.3 (7.8-14.8)	9.3 (6.9-11.6)

Lifetime	33.4 (28.9-37.8)	38.7 (33.3-44.1)	32.8 (27.8-37.8)	37.0 (30.8-43.3)	33.7 (29.5-37.9)

Suicide attempt					

12-month	2.5 (1.5-3.5)	3.8 (1.3-6.2)	2.1 (1.1-3.1)	4.4 (2.0-6.9)^#^	2.6 (1.6-3.6)

Lifetime	19.9 (16.0-23.7)	28.7 (20.8-36.7)*	18.7 (14.4-23.0)	26.9 (21.7-32.0)^#^	20.5 (16.8-24.1)

*Statistically significant (*p *< .05) difference between men and women. ^#^Statistically significant (*p *< .05) difference between non-Aboriginal and Aboriginal persons

As expected, there was a strong association between suicidal ideation and suicide attempt. More than half (57.5%; 95% CI 51.0%-64.0%) of participants reporting lifetime suicidal ideation reported a lifetime suicide attempt. Almost all participants (94.8%; 95% CI 91.6%-97.9%) reporting a lifetime suicide attempt also reported lifetime suicidal ideation.

### Correlates of lifetime suicidal ideation

In univariate analyses, lower education levels, childhood experiences of out-of-home care, harmful alcohol use and regular illicit drug use in the 12 months prior to incarceration were significantly associated with increased odds of lifetime suicidal ideation. None of these variables retained significance in the multivariate model. In the multivariate model, older age, having a violent offence as one's most serious offence, a history of TBI, BDI scores indicative of moderate to severe depression, and self-harm without suicidal intent were all associated with significantly increased odds of lifetime suicidal ideation (Table 2).

**Table 2:** Correlates of lifetime suicidal ideation in NSW prisoners

	Univariate		Multivariate	
	
	OR (95% CI)	*p*	AOR (95% CI)	*p*
Female	1.26 (0.93-1.70)	.13		

Aboriginal	1.20 (0.85-1.70)	.29		

Age > 33 years	1.55 (1.13-2.14)	.007	2.20 (1.56-3.12)	< .0001

Year 10 education or above	0.64 (0.47-0.89)	.007	0.89 (0.60-1.33)	.57

Out-of-home care aged < 16	1.78 (1.33-2.39)	< .0001	1.23 (0.91-1.66)	.18

Juvenile detention history	1.34 (0.96-1.87)	.08		

Parental incarceration	0.93 (0.57-1.52)	.77		

Remand inmate	1.10 (0.66-1.82)	.73		

Violent offender	1.62 (1.17-2.25)	.004	1.62 (1.13-2.32)	.008

Harmful alcohol use	1.65 (1.21-2.23)	.001	1.26 (0.84-1.88)	.27

Regular illicit drug use	1.39 (1.08-1.79)	.01	1.26 (0.92-1.71)	.15

Traumatic brain injury	2.00 (1.34-3.00)	.0007	1.67 (1.08-2.58)	.02

Moderate/severe depression	3.36 (2.59-4.35)	< .0001	3.06 (2.25-4.17)	< .0001

Self-harm without suicidal intent	10.93 (6.85-17.47)	< .0001	9.81 (5.58-17.23)	< .0001

OR = Odds ratio. AOR = Adjusted odds ratio (adjusted for all other factors in the multivariate model).

### Correlates of suicide attempt among participants reporting suicidal ideation

As shown in Table 3, in univariate analyses, a history of out-of-home care, parental incarceration, TBI, and moderate/severe depression were associated with suicide attempt among participants who reported suicidal ideation. In the multivariate analysis, none of these variables retained statistical significance.

**Table 3:** Correlates of lifetime suicide attempt among NSW inmates reporting lifetime suicidal ideation

	Univariate		Multivariate	
	
	OR (95% CI)	*p*	AOR (95% CI)	*p*
Female	1.59 (0.72-3.52)	.25		

Aboriginal	1.60 (0.96-2.66)	.07		

Age > 33 years	0.78 (0.45-1.33)	.36		

Year 10 education or above	0.87 (0.54-1.39)	.55		

Out-of-home care aged < 16	2.20 (1.29-3.74)	.004	1.82 (0.98-3.36)	.06

Juvenile detention history	1.72 (0.92-3.21)	.09		

Parental incarceration	2.38 (1.24-4.58)	.009	2.05 (0.99-4.21)	.05

Remand inmate	1.36 (0.85-2.17)	.20		

Violent offender	1.12 (0.68-1.86)	.67		

Harmful alcohol use	0.93 (0.50-1.73)	.82		

Regular illicit drug use	1.15 (0.63-2.09)	.67		

Traumatic brain injury	1.76 (0.99-3.08)	.05	1.55 (0.88-2.73)	.13

Moderate/severe depression	1.58 (0.99-2.48)	.05	1.38 (0.85-2.26)	.20

Self-harm without suicidal intent	1.46 (0.87-2.45)	.16		

OR = Odds ratio. AOR = Adjusted odds ratio (adjusted for all other factors in the multivariate model).

## Discussion

### Population prevalence estimates

Suicidal ideation and suicide attempts are common among people in NSW prisons. An estimated 33.7% of prisoners in NSW have had suicidal thoughts during their lifetime, while 20.5% report suicide attempts. In comparison, 13.3% of Australian adults in the 2007 National Survey of Mental Health and Wellbeing reported lifetime suicidal ideation, and 3.2% reported a suicide attempt [28]. As in the general population, a greater proportion of women than men had attempted suicide; however, prevalence was greatly elevated above that seen in the general population for both women (28.7% vs. 4.4%) and men (19.9% vs. 2.1%) [28]. The finding that women prisoners in particular are vulnerable to suicide attempts highlights the need for gender-specific suicide prevention interventions. Women in prison frequently have extensive trauma histories and multiple psychiatric co-morbidities [29-31]; addressing the range of traumas experienced by women in prison is likely an important component of effective suicide prevention for this group [24,30,31].

A significantly greater proportion of Aboriginal than non-Aboriginal inmates had attempted suicide. Although this is in line with general population data demonstrating higher rates of completed suicide among Aboriginal than non-Aboriginal Australians [18], this result nevertheless contrasts with the findings of a previous study in the same prison system that found no relationship between Aboriginality and suicide attempts [19]. There are several potential explanations for this discrepant finding. There may have been genuine changes over time in the prevalence of suicidality among Aboriginal and/or non-Aboriginal inmates. Alternatively, the use of a telephone interview in the current study, as opposed to a face-to-face interview in the earlier study, may have influenced responding. Finally, the earlier study derived its sample by combining a subset of a random sample with new receptions to prison [19]. This sampling strategy may have obscured differences in suicidality between Aboriginal and non-Aboriginal inmates that became apparent when using a more strictly selected sample.

Suicide among Australian Aboriginal people is a complex phenomenon, influenced by individual and community-level psychological distress, inter-generational trauma and social marginalisation [32,33]. Although research is limited, there are suggestions that compared to non-Aboriginal persons, mental illness has less influence, and situational factors more influence, on Aboriginal suicide [32,34]. Culturally appropriate suicide risk assessments and interventions are therefore needed, as generic approaches may not identify Aboriginal prisoners at risk, or address their specific risk factors.

### Correlates of suicidal ideation

Despite the greater prevalence of suicide attempts among women and Aboriginal inmates, gender and Aboriginality were not in themselves risk factors for suicidal ideation. Rather, psychiatric factors - depression and self-harm without suicidal intent - were the strongest correlates of suicidal ideation, highlighting the importance of providing adequate mental health services in prison.

A history of TBI was associated with increased odds of suicidal ideation. An association between TBI and suicidality has previously been reported in general population samples [35]. As with other factors associated with suicidal ideation, the prevalence of TBI is elevated in prisoner populations [24]. It is thought that TBI increases the risk of suicide via two pathways; the injury itself may act as a stressor, precipitating suicidal ideation, and increased impulsivity as a result of the injury may increase the tendency to act on suicidal thoughts [36]. Impulsivity may also underlie the observed association between suicidal ideation and violent offending [15].

Although problematic substance use has been associated with suicidal ideation in general population samples [37], in this prisoner sample neither hazardous alcohol use nor regular pre-incarceration illicit drug use were associated with suicidal ideation in the multivariate analysis. At least one other study of suicidal ideation in prisoners has reported a lack of association with alcohol and drug use [13]. The high prevalence of problematic substance use among prisoners may mean that it is not a useful predictor of suicidality in this population.

### Suicide attempts among ideators

Over half of participants who reported suicidal ideation also reported a suicide attempt, confirming the importance of suicidal ideation in identifying prisoners most at risk of suicide attempt. Suicide attempt in the absence of suicidal ideation was rare. It could be argued that a suicide attempt cannot occur in the absence of suicidal ideation [38]; however, it is possible that some participants perceive their suicide attempt/s as impulsive acts that occurred in the absence of formal thoughts about suicide.

The analysis reported in Table 3 was aimed at identifying factors that may be clinically useful in identifying who, of the large number of people in prison reporting suicidal ideation, may be more likely to attempt suicide. In the multivariate analysis, none of the assessed factors were statistically significant; however, the odds ratios associated with parental incarceration and out-of-home care as a child neared significance. These factors may be indicators of childhood trauma, which itself is strongly associated with suicide attempts [39]. Parental incarceration and out-of-home care may be clinically relevant indicators of risk of suicide attempt, but a more direct measurement of childhood trauma would allow for better understanding of this relationship.

Research on general population samples has noted that although mental illness is highly prevalent among people reporting suicidal ideation, a mental illness diagnosis is of limited utility in predicting who, of people with suicidal ideation, will go on to attempt suicide [40]. Our results confirm that this is also the case among prisoners. Moderate/severe depression was significantly associated with increased risk of suicidal ideation, but of participants reporting suicidal ideation, moderate/severe depression was not associated with suicide attempt when other factors were controlled. Similarly, although strongly associated with suicidal ideation, a history of self-harm without suicidal intent was not associated with having attempted suicide. This was surprising, as self-harm is generally associated with an increase in subsequent suicide attempts and completed suicide [41]. However, a general population survey in the United Kingdom reported some separation between self-harm without suicidal intent and suicidal attempts [38]; our results suggest that among prisoner populations, self-harm and suicide attempts may indeed be distinct behaviours, and that self-harm may not be indicative of risk of suicide attempt. That said, self-harm remains a behaviour of serious concern to mental health clinicians in prisons, as self-inflicted injuries can be fatal even in the absence of suicidal intent. These results suggest that self-harm without suicidal intent, and suicidal ideation and attempt, are at least in part separate issues and may therefore require discrete risk assessment and intervention strategies.

### Limitations

The retrospective, cross-sectional nature of the survey prevented us from examining the ordering of onset of suicide risk factors (e.g. psychiatric disorders), suicidal ideation and suicide attempts. Understanding of the temporal relationships between these factors may assist in more clearly delineating which prisoners who experience suicidal ideation will go on to attempt suicide. Large prospective studies would be most suitable for obtaining such data; in their absence, future cross-sectional studies of prison suicide should attempt to obtain data regarding onset of risk factors, suicidal ideation and suicide attempts.

It is important to bear in mind the potential for bias as a result of stigma and sensitivity around suicide, and the self-report nature of the data. It is not possible to determine the extent to which this may have affected the results; under-reporting of suicidal behaviours would contribute to more conservative findings.

## Conclusions

The high prevalence of suicide risk factors among prisoner populations complicates the task of identifying which prisoners are most at risk of attempting suicide. We found that among prisoners expressing suicidal ideation, parental incarceration was associated with having made a suicide attempt, but depressive symptoms and self-harm were not. Further work is required to determine particular patterns of risk factors that heighten the likelihood of a suicide attempt. Prospective studies encompassing entire prisoner populations would assist in more clearly delineating associations between risk factors for suicide, and suicide attempt, among prisoners.

## Competing interests

SL, DI and DG are current employees, and LT and CO are past employees, of Justice Health, which funded the Inmate Health Survey.

## Authors' contributions

SL conceived of the research questions, conducted the statistical analysis and drafted the manuscript. LT helped conduct the Inmate Health Survey and helped to draft the manuscript. DI led the conduct of the Inmate Health Survey and helped to draft the manuscript. CO helped to draft the manuscript. DG helped conduct the Inmate Health Survey and helped to draft the manuscript. All authors read and approved the final manuscript.

## Pre-publication history

The pre-publication history for this paper can be accessed here:

http://www.biomedcentral.com/1471-2458/12/14/prepub
